# Real-time vehicle control via edge cloud sensor fusion and CNN based perceptron

**DOI:** 10.1016/j.mex.2025.103779

**Published:** 2025-12-24

**Authors:** Sumukh Chaurasia, Parambrata Sanyal, Gagandeep Kaur, Satvik Barhanpure, Kshitij Bhele, Amol D. Wable, Suhashini Awadhesh Chaurasia, Rutuja Rajendra Patil, Devika Verma

**Affiliations:** a Symbiosis Institute of Technology, Nagpur Campus, Symbiosis International (Deemed University), Pune, India; b Sanjivani College of Engineering, Kopargaon, India; c Tulsiramji Gaikwad-Patil College of Engineering and Technology, Nagpur, India; d Department of Computer Engineering, MIT, Academy of Engineering, Alandi, Pune, India; e Vishwakarma Institute of Technology, Pune, Maharashtra, India

**Keywords:** Deep learning (DL), Intelligent transport system (ITS), Internet of things (IoT), Artificial intelligence (AI), Smart vehicle

## Abstract

Reliable real-time vehicle control is essential for intelligent transport systems where accurate perception and decision-making depend on fast sensor data processing. This study developed a hybrid edge–cloud method integrating deep learning with Internet of Things (IoT) sensor fusion for adaptive vehicle control. Ultrasonic range data were combined with convolutional neural networks (CNNs) to enable object detection, stopping-time prediction, and braking control under varying environmental conditions. The CNN-based model was trained and evaluated under normal and simulated adverse driving scenarios. Results indicated strong performance with R² = 0.99 under normal and 0.98 under adverse conditions, and a mean squared error (MSE) of 0.0085. Average inference latency is 110–116 ms on Jetson Nano and 210–230 ms on Raspberry Pi, confirming suitability for real-time deployment on edge hardware.

The hybrid edge–cloud method enables adaptive, real-time vehicle control through IoT sensor fusion.

CNN-based perception enhances prediction accuracy and operational safety under variable driving conditions.

Demonstrates feasibility of deep learning deployment on low-cost edge devices for intelligent transport applications.

Thus, integrating deep learning with IoT-enabled sensors on an edge–cloud platform provides a reliable and scalable pathway toward safe, adaptive, and efficient vehicle control in intelligent transportation systems.

## Graphical abstract

**Figure:**
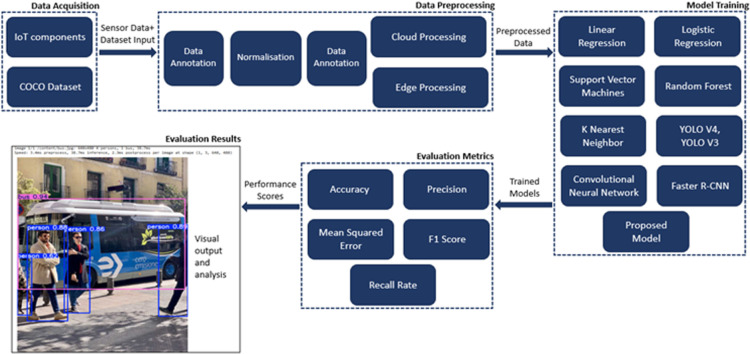

**Specifications Table**

**Table:** 

Subject area	Computer Science
More specific subject area	Deep Learning and IoT
Name of your method	TensorFlow based Convolutional Neural Networks and IoT sensors
Name and reference of original method	• Linear Regression [1] • Logistic Regression [2] • Support Vector Machines [3] • Random Forest [4] • K Nearest Neighbour (KNN) [5] • YOLO v4 [6] • YOLO v3 [7] • Convolutional Neural Networks (CNN) [8] • Faster R-CNN [9]
Resource availability	GitHub, Dataset: Object-detection-and-prediction/Dataset at main · 9SERG4NT/Object-detection-and-prediction

## Background

With the rapid growth of intelligent transport system (ITS), integrating modern technologies can make them safer and more reliable [10]. Real-time object identification is very viable for the accurate functioning of braking and speed control. This is used when preventing an accident and safeguarding the passengers; this has gained a lot of attention in the recent past [11,12]. Researchers have highlighted how accident detection systems can contribute to society by providing timely aid and preventing further loss of lives [13]. The motivation stems from the growing demand for safer, more efficient ITS that can reduce road accidents and traffic inefficiencies, whereas traditional driver-assistance systems often fail to provide timely and reliable object detection [14,15]. “Global status report on road safety says road traffic deaths have fallen slightly to 1.19 million per year with the minor efforts made to improve road safety, underlining the need for intelligent systems that minimize human intervention and improve decision-making in real time” (World Health Organisation, 2023) [16]. Deep learning-based solutions appear to be a more feasible option because they can learn complex patterns from diverse datasets [17]. Intelligent perception systems capable of operation under real-world driving conditions are a pressing requirement [[18], [19], [20]]. This work is aimed at filling the gap between traditional vehicular automation and fully autonomous driving through IoT-based sensor fusion and deep learning powered object detection for a smarter, safer, and more efficient transportation future.

Ahmed et al. proposed a smart IoT-enabled deep learning-based end-to-end 3D object detection system for real-time autonomous driving applications, based on the YOLOv3 model (originally developed for 2D object detection and then adapted for 3D object detection by incorporating both RGB image data and LiDAR-generated point cloud data) [21]. Liang et al. proposed Edge YOLO: A cooperative edge cloud system using reconstructive convolutional neural networks for autonomous vehicles, as an object detection system for vehicles [22]. Alam et al. presented a smart city project system that uses Ultrasonic as well as Camera Sensors to achieve object recognition and detection for collision-free navigation, along with distance measurement between the vehicle and obstacles. The system also integrates IoT technology, using a GPS and Wi-Fi module to continuously send location data to a mobile device for tracking purposes. Also, vehicle can be controlled through voice commands using Bluetooth, which increases user interaction [23]. The approach to an ITS put forward by Sharma and Garg is to enhance the safety of roads, prevent road accidents using connected vehicle technology, and YOLOv4 real-time computer vision [24].

Based on machine learning and reinforcement learning, Amrith et al. proposed an AI-based accident detection and emergency response system to enhance efficiency in reporting accidents and the assessment of the severity [25]. Li et al. proposed an Intelligent Visual Internet of Things (IVIoT) surveillance system, which integrates techniques of computer vision and pattern recognition to enhance smart monitoring for vehicle management on road [26]. Alasmari et al., in their research aims to propose an improved Metaheuristics technique using Deep Learning based object detectors for Intelligent Control in Autonomous Vehicles (IMDLOD-ICAV), with the application of enhancing the detection of objects and decision making for autonomous vehicle control [27]. The framework by Jiang et al. for the object detection from the (V-IoV) incorporates edge AI while addressing problems, such as the similarity of objects, and background noise [28]. Mia and Amini proposed a hybrid privacy preserving algorithm to improve the security of Federated Learning (FL) in ITS, particularly for object detection tasks [29]. Sabri et al. designed an IoT-based intelligent transportation system focused on preventing accidents and taking post-crash measures with the prime objective of improving road safety [30] (Table 1).

**Table 1:** Comprehensive literature survey table.

Publication	Proposed Model	Methodology	Dataset and Technique Used	Performance	Application
[21]	Object Detection in Autonomous Vehicles	YOLOv3 for 2D and 3D object detection	RGB image data, LiDAR	96 % accuracy for 2D, 97 % accuracy for 3D	Smart Vehicles, Environment Perception
[22]	Edge YOLO for Autonomous Vehicles	Edge-cloud cooperation, Reconstructive CNNs	NVIDIA Jetson, COCO2017, KITTI	26.6 FPS, 47.3 % mAP	Autonomous Vehicles, Energy-Efficient Object Detection
[23]	IoT integrated Autonomous Vehicles	Ultrasonic Sensors, Cameras, IoT components	GPS, Wi-Fi, Bluetooth	-	Intelligent Transport Systems
[24]	ITS and YOLO for Traffic Safety	YOLO-v4 based real time object detection, IoT	Cloud based Geospatial Database	0.9777 mAP, 74.26 FPS	Accident Prevention, Accident Safety
[25]	ML based Accident Detection and Emergency Response	K-means clustering, SVM, (BFS-A*) Search Algorithm	Sensor Data	Fast Detection and Routing	Emergency Response, Accident Management
[26]	IVIoT surveillance for Vehicle Management	Computer Vision, Pattern Recognition	Image Processing	85.8 % Real time Tracking Accuracy	Vehicle Tracking, Urban Traffic Management
[27]	IMDLOD-ICAV for Autonomous Vehicles	Deep Learning Based Object Detection	RetinaNet, Elman Neural Network, IDFA	98.38 % Accuracy	Autonomous Vehicle Object Detection
[28]	Edge AI Framework, V-IoV	Abductive Learning Algorithm, YOLO	Symbolic and AI Blockchain	High Accuracy	Vehicle Systems, V-IoV Systems
[29]	Privacy Preserving Federated Learning	Hybrid Algorithm	Edge Devices	High Accuracy	Secure ITS, Object Detection
[30]	IoT enabled Intelligent Transport Systems	IoT enabled Vehicle Communication	IoT Based Real Time Data	Improved Road Safety	Traffic Safety, Accident Prevention

## Method details

The proposed approach uses a hybrid edge-cloud deep learning framework for real-time vehicle control by fusing ultrasonic sensor information with camera perception. Three functional layers are used in harnessing the process: sensor acquisition, edge-cloud data processing and actuation control, which guarantee quick response, flexibility and energy-efficient performance. Combining TensorFlow-based CNN inference on the edge device with periodic retraining on the cloud is carried out within the system to provide long-term adaptability.

**Fig. 1:**
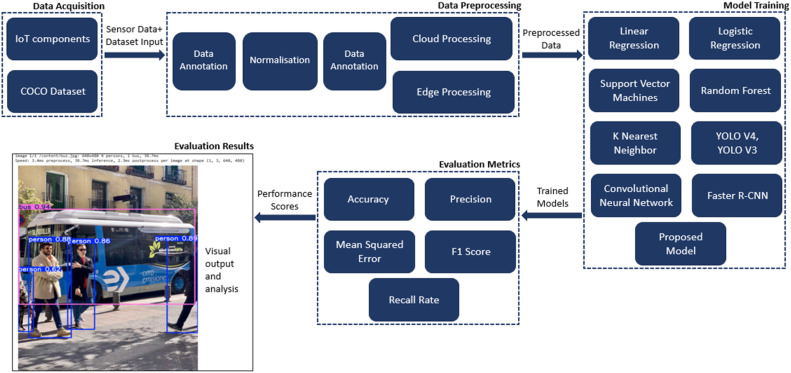
Structured workflow for proposed model.

**Fig. 2:**
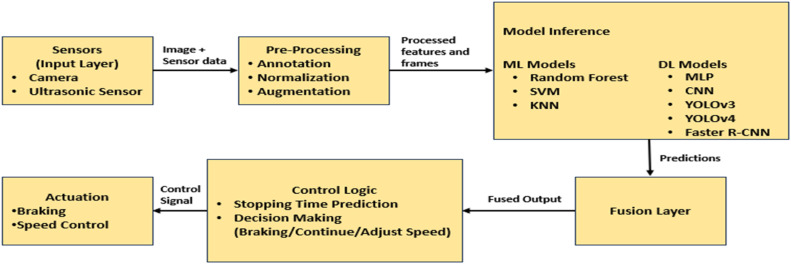
Proposed model architecture.

The novelty of this work lies in its edge-cloud hybrid processing framework, which enables highly efficient real-time object detection, multi-layered sensor fusion for precise vehicle control, and deep learning-based decision-making to enhance the safety of autonomous vehicles. Unlike prior YOLO- or CNN-only approaches, the proposed system integrates edge inference with cloud-based retraining and updates, offering both low latency and adaptability. In particular, adaptability is achieved at two-time scales: (i) short-term, on-device adaptation in control thresholds using recent sensor/state buffers and (ii) long-term model adaptation via periodic cloud retraining using edge-collected data. The workflow consists of sensor input, preprocessing, inference (CNN/MLP), fusion, and actuation, providing a structured pipeline for reliable control. The measured inference latency of the deployed CNN model ranges from 110 to 116 ms, thus proving the feasibility of real-time operation on commodity hardware (Figs. 1, 2).

The proposed model uses a layered architecture approach for real-time data processing and data fusion collected from several sensors, also applying the local and cloud resources. The different layers include:

Sensor Layer: This layer includes a number of in-car cameras and an ultrasonic sensor, together with other sensors that feed real-time environmental information. Ultrasonic sensors would measure distances to objects in proximity, relevant, and necessary information. Data Processing Layer: This layer carries out two important duties: (i) Edge Processing: Raspberry Pi(s), NVIDIA Jetson, etc. use TensorFlow Lite to perform real-time object detection and data preprocessing, which reduces latency. (ii) Cloud Processing: Different computations, such as model retraining, model updates, and large-scale data handling, are performed on the cloud server. The cloud server also receives feedback (inference logs and anonymized samples) from edge devices.

Control Layer: This layer receives input from the object detection layer and implements the vehicle’s speed-control and braking logic. The decisions at this layer help respond to observed threats in real time. Short-term adaptivity — the control logic maintains a short temporal buffer of recent sensor readings and detection outputs. Control thresholds (e.g., braking intensity and warning thresholds) are adjusted dynamically according to detected object type, relative velocity, and recent detection confidence, rather than relying on fixed static thresholds. This allows context-sensitive actions (gradual deceleration for slowly approaching obstacles versus emergency braking for imminent collisions) and reduces false alarms under noisy sensor conditions*.*

## Data handling

Data for evaluation were collected from multiple sensors during controlled tests that simulated realistic car-driving scenarios. The following datasets were utilized:

Custom Dataset: The dataset was recorded using color cameras and ultrasonic range finders during natural test drives across urban, suburban, and rural environments. It also contains annotations of objects like pedestrians, cyclists, vehicles and obstacles.

Public Dataset: For the initial model training and validation, standard datasets such as COCO and KITTI were employed to supplement the labeled data and strengthen model generalization. Preprocessing Steps: The data preprocessing phase involved the following steps: (i) Data Annotation: Collected image frames were annotated with bounding boxes to identify objects of interest, using tools such as Label Image. (ii) Normalization: All images were scaled to the interval [0,1] to improve convergence of training sessions, as was needed by the algorithm. (iii) Data Augmentation: To enhance the data size and sampling process, and to make the models more immune to different situations, random rotation, translation, scaling, and flipping were performed.

## Model training

The model training process is further divided into the following steps:

Framework: The TensorFlow deep-learning framework (version 2.x) was adopted for model development and training due to its flexibility, robust deep-learning primitives, and scalability for handling large datasets.

Dataset: The model has been trained using the COCO dataset, which contains several images with multiple object categories, for optimized output. This dataset is very crucial for performance improvement in terms of putting it into practice in real environments.

Loss Function: To assess the network’s response, we have used the classification loss function (categorical-cross entropy) on top of the normal bounding box regression loss to refine the processing of the network in the localization of objects. This two-loss strategy enables the model to simultaneously learn object localization and class prediction, thereby improving detection precision. The prediction framework was designed with a clear distinction between independent and dependent variables. The independent variables are:

Vehicle Speed: The instantaneous forward velocity of the vehicle, measured in meters per second.

Trajectory Angle: The directional angle of motion relative to a reference axis, measured in degrees.

The dependent variable is:

Future Position: The predicted displacement of the next spatial coordinate of the vehicle, estimated based on the current kinematic state.

Both the speed and trajectory angle were obtained from the sensor data (camera stream combined with simulated kinematic sensor readings). The ground truth future position was generated from controlled simulation runs, providing labeled data for supervised learning.

Model Architectures

MLP (Multilayered Perceptron): build_mlp_model()

Number of hidden layers: 2

Units per hidden layer:

Hidden layer 1: 64 units (Dense, ReLU)

Hidden layer 2: 32 units (Dense, ReLU)

Output layer: Single neuron with linear activation to predict displacement.

CNN (Convolutional Neural Networks): build_cnn_model()

Number of hidden layers: 2 (1 convolutional + 1 dense hidden layer)

Units per hidden layer:

Convolutional layer: 64 units (kernel size = 2, ReLU)

Dense layer 2: 32 units (ReLU)

Output layer: Linear neuron producing predicted displacement.

Table 2 below summarizes the independent/dependent variables and the network configurations used in this study. Table 3 shows the stepwise summary of the proposed vehicle control framework using edge–cloud sensor fusion

**Table 2:** Model architectures with independent/dependent variables and hidden layer configurations.

Model	Independent Variables	Dependent Variable	Hidden Layers	Units per Hidden Layer
MLP	speed, angle	future_position	2	Layer 1: 64, Layer 2: 32
CNN	speed, angle	future_position	2 (1 Conv + 1 Dense)	Conv1D: 64 filters, Dense: 32

**Table 3:** Summary of methodological pipeline for proposed real-time vehicle control system.

Stage	Input/Process	Technology/Framework	Output/Result
Data Acquisition	Camera frames, ultrasonic sensor data	IoT sensors	Raw multimodal data
Preprocessing	Annotation, normalization, augmentation, synchronization	Python, OpenCV	Structured dataset
Model Training	CNN (TensorFlow 2.x), bounding-box regression	TensorFlow, COCO dataset	Trained CNN model
Edge Inference	On-device object detection and control	TensorFlow Lite, TensorRT	Real-time control action
Cloud Retraining	Model updates using edge data	Cloud server, TensorFlow	Adaptive model refinement
Deployment & Control	Edge–cloud integrated feedback	IoT edge nodes, actuator control	Dynamic braking/speed adjustment

### Model implementation

#### System overview

The proposed system is designed for effective control of vehicle movement using distance sensors for measuring distances, and an object recognition model trained by TensorFlow. The system decides whether to accelerate, maintain speed, or slow down, depending on the objects seen and recognizing their distance. Moreover, the time that would be taken for the vehicle to stop at a particular speed would also be computed by the system.

#### Hardware requirements

The hardware requirement involves several components. A list of them includes: (i) An ultrasonic sensor, which will be used to detect the distance to the surroundings in relation to objects like cars and people. (ii) A high-quality camera will be used to capture pictures for object detection in real time. (iii) The processing unit will incorporate an edge device, for instance, NVIDIA Jetson Nano or Raspberry Pi, and would also involve TensorFlow model for sensor data analysis. This setup will be connected with the vehicle’s onboard control system to allow proportional control of acceleration and braking.

#### Data collection

During data collection, data were recorded by capturing distance measurements from the ultrasonic sensors and images from the camera while the robot was in motion. Recording the speed of the vehicle during this process is vital to relate to distance calculations and object recognition data. For the custom dataset, images containing cars, trucks, and other objects were annotated using appropriate labeling tools for object detection tasks.

#### Data preprocessing

Data preprocessing contains several stages for the preparation of the images and distance data for the model. For image preprocessing, all images were normalized to a range between 0 and 1 and resized to a uniform dimension of 224×224 pixels to ensure compatibility with the model input. Additionally, distance measurements obtained from ultrasonic sensors were rectified and standardized before being used in the machine learning model. Data augmentation improved performance, increasing R^2^ from 0.985 to 0.990.

#### Final model

The final object detection model was developed using TensorFlow (through CNN). An ultrasonic sensor detects the object, and the camera passes the data to our model, which predicts the time taken by the vehicle to stop at the speed at which it is running, predicts that the vehicle should speed up, slow down, or maintain speed. The TensorFlow-based model detects whether the image contains a car, truck, animal, or other object.

## Model operation and results

The working section is divided into three scenarios as described below. When the system initializes, an LED indicator turns on. The ultrasonic sensor is used to detect nearby objects, and TensorFlow identifies the detected objects as shown in Fig. 5. This information is then passed to the pre-trained model, which predicts the time required to stop the vehicle at the current speed. Furthermore, it also predicts whether to accelerate, decelerate, or maintain the speed so that the destination can be reached as efficiently as possible without unnecessary delay.

Figures below 3,4,5 shows an ultrasonic sensor, LED, LCD screen and a microcontroller. Green colored ball on the radar depicts an object. The simulator-based execution has been performed in the TINKER CAD platform.

### Case 1: object is far

**Fig. 3:**
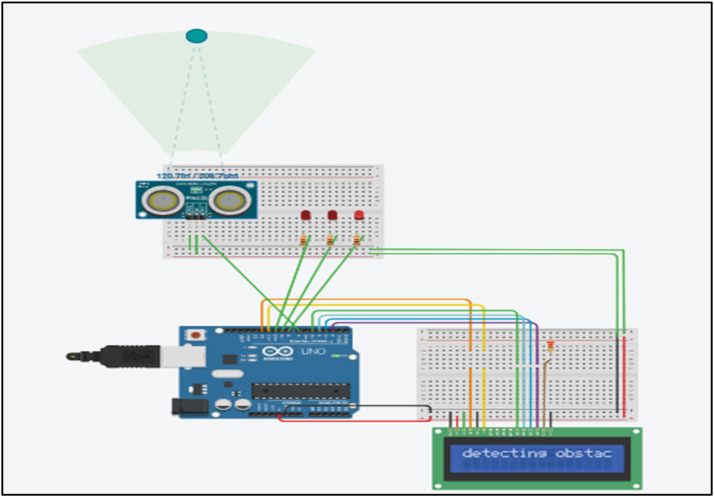
Simulation output for case 1 where the object is far from the sensor.

Simulation results in Fig. 3 illustrate the effect of an object being positioned far from the sensing device. When the object is far from the sensor, the model detects it. When an obstacle is detected outside the collision zone, the display panel notifies this by showing the message 'detecting obstacle'.

### Case 2: object is closer

**Fig. 4:**
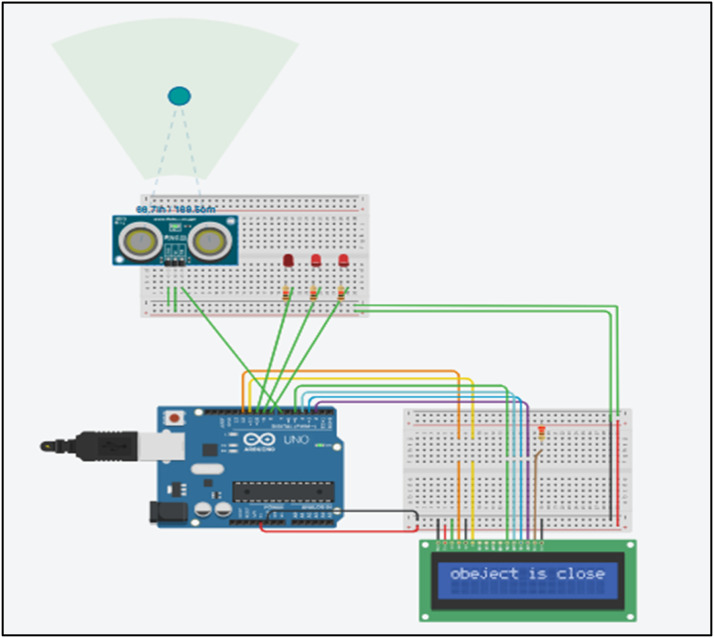
Simulation output for case 2 where the object is closer to the sensor.

Simulation execution in Fig. 4 demonstrates the effect of an object positioned closer to the sensing device. As the object approaches the sensor, the model detects its presence and assesses the likelihood of collision. When a nearby object is identified, two red LED lights illuminate and the display panel displays the message 'object is close'.

### Case 3: object is about to collide

**Fig. 5:**
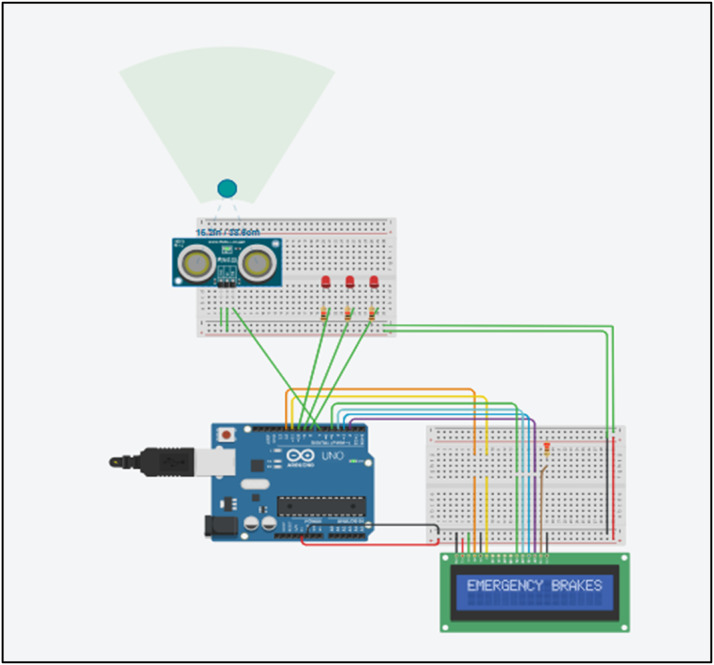
Simulation output for case 3 where the object is about to collide.

Fig. 5 below shows the execution on the simulator showing the effect of object when it is about to touch the sensing device. When the object is so close that it has a high probability of colliding, the model detects it. As soon as the object is detected, all three red LED lights glow and the display panel shows the message “EMERGENCY BRAKES”.

**Fig. 6:**
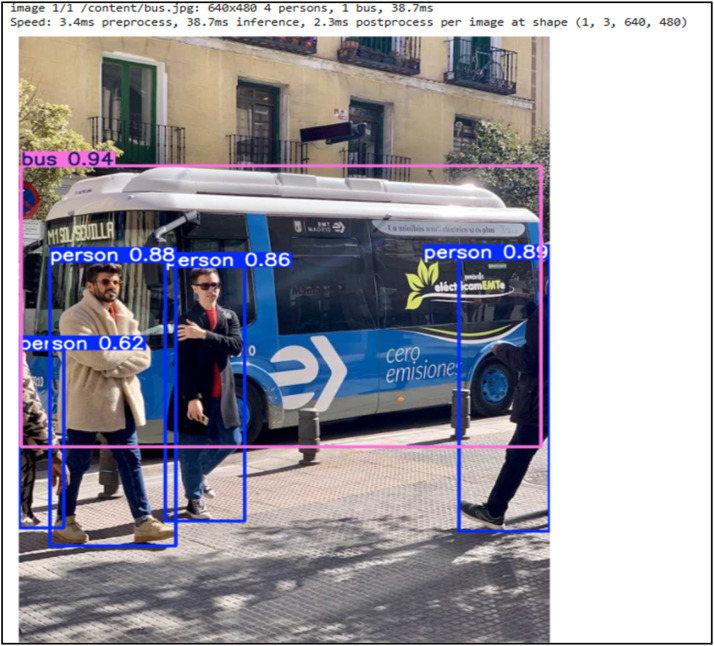
Object detection by TensorFlow with accuracy.

Fig. 6 below shows the output of the TensorFlow model. The model displays the name of the object and also displays probability of the object. Following is the output predicted by the trained model along with the accuracy.

**Fig. 7:**
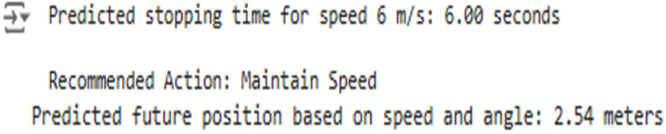
Python code snippet output block.

Fig. 7 below shows output for predicted stopping time for speed 6 m/s: 6:00 s. Recommended Action: Maintain Speed. Predicted future position based on speed and angle: 2.54 m.

## Evaluation results

**Fig. 8:**
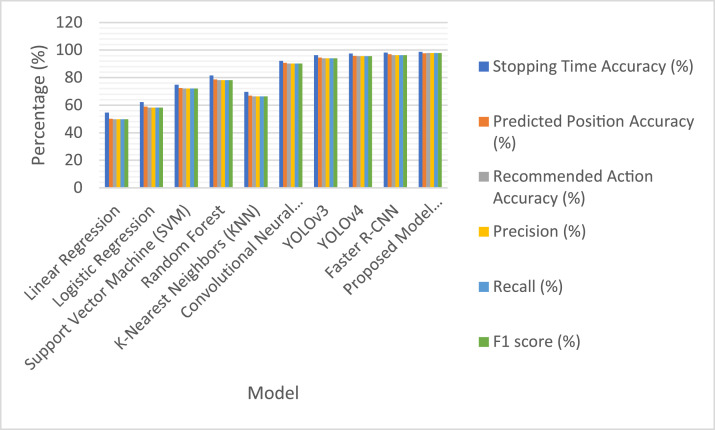
Performance comparison of different models across key metrics.

Table 4 and Fig. 8 compare various machine learning and deep learning models using eight performance metrics. Traditional models are Linear Regression and KNN, which have limited accuracy and a stopping time accuracy lower than 70 %. Deep learning models include CNN and YOLO variants that demonstrate very high performances, with up to 95.8 % predicted position accuracy in YOLOv4. The proposed model has outperformed all of them with top scores in most metrics, showing that it is well balanced and optimized in its control strategy. It is important to note the substantial improvement of the current approach in recommended action accuracy, which outperforms earlier CNN-based models.

**Table 4:** Evaluation results of different models.

Model	Stopping Time Accuracy (%)	Predicted Position Accuracy (%)	Mean Squared Error (MSE)	Recommended Action Accuracy (%)	Precision (%)	Recall (%)	F1 score (%)
Linear Regression	54.6	50.2	0.256	49.8	49.8	49.8	49.8
Logistic Regression	62.3	58.9	0.197	58.2	58.2	58.2	58.2
Support Vector Machine (SVM)	74.8	72.4	0.132	72.1	72.1	72.1	72.1
Random Forest	81.5	78.6	0.095	78.2	78.2	78.2	78.2
K-Nearest Neighbors (KNN)	69.7	66.8	0.142	66.3	66.3	66.3	66.3
Convolutional Neural Network (CNN)	92.1	90.7	0.048	90.2	90.2	90.2	90.2
YOLOv3	96.3	94.5	0.028	94.1	94.1	94.1	94.1
YOLOv4	97.5	95.8	0.019	95.5	95.5	95.5	95.5
Faster R-CNN	98.1	96.9	0.012	96.3	96.3	96.3	96.3
Proposed Model (TensorFlow-based CNN + IoT Sensors)	**98.7**	**97.6**	**0.0085**	**97.9**	**97.9**	**97.9**	**97.9**

The models were tested under adverse conditions to access robustness beyond controlled simulations. Noise was injected into sensor input streams to simulate fog, rain, and environmental disturbances. Table 5 below presents the evaluation results of the different models under adverse conditions.

**Table 5:** Evaluation results under adverse conditions.

Model	Condition	MSE	R²	Latency (ms)
MLP	Normal	0.012	0.985	85– 95
MLP	Adverse	Higher than normal	Lower than normal	90– 100
CNN	Normal	0.0085	0.990	100– 116
CNN	Adverse	Slightly higher than normal	0.980	110– 116
Random Forest	Normal	Higher than MLP/CNN	0.970	8– 16
Random Forest	Adverse	Higher than normal	Lower than normal	10– 20

## Method validation

The proposed TensorFlow-based CNN model integrated with IoT sensors outperformed all baseline models, with the best accuracy of 98.7 %, lowest MSE of 0.0085, and the highest precision, recall, and F1-score, which were all 97.9 %. When compared with other traditional and deep learning models, such as YOLOv4 and Faster R-CNN, it showed better performance for real-time control, hence proving that the combination of deep learning with IoT works perfectly for intelligent vehicle decisions. In order to further validate the real-time feasibility on resource-constrained devices, the trained model was deployed on a Raspberry Pi 4 (4GB RAM) and an NVIDIA Jetson Nano (4GB). Inference latency was measured on video input streams under different traffic scenarios. On the Raspberry Pi, the average detection latency per frame was 210–230 ms, approximately 4–5 FPS. On the Jetson Nano, after TensorRT optimization, the latency was reduced to 110–116 ms, enabling real-time performance at 8–9 FPS. The memory utilization on the Jetson Nano remained below 70 %, confirming its efficiency for edge deployment. These measurements include preprocessing and non-maximum suppression (NMS) overhead and were averaged over multiple trials to ensure stability. The results indicate that the proposed edge-cloud hybrid design is not only accurate but also practical for real-world ITS applications, in contrast to previous YOLO-based solutions that predominantly rely on high-end GPUs. **Sensor Calibration and Environmental Noise** Accurate sensor calibration is important for assurance of reliable input data for the control of autonomous vehicles. Environmental noise strongly affects both accuracy and model performance. Camera sensors are prone to weather variations, such as fog, rain, and glare, which obscure visibility and reduce the degree of confidence in detection. On the other hand, while ultrasonic sensors are not influenced by light, changes in rain, temperature, or humidity may cause misreadings.

The different challenges can all be addressed by the sensor fusion that merges the visual and ultrasonic data to leverage each of the strengths in maintaining consistent object detection and control. From a deployment perspective, the edge-cloud architecture raises issues of data security and confidentiality. There is fortification of security with encrypted transmission, anonymization on-device, and signed model updates, while both the data and adversarial attacks are guarded against. Reliability is improved through sensor health monitoring, fault detection and fail-safe modes that trigger controlled operation upon the failure of any sensor. Integrated strategies assure data accuracy, system robustness and real-time responsiveness, meeting the requirements for safety in adaptive vehicle control within intelligent transport systems.

### Security, privacy and reliability constrains

The hybrid edge-cloud framework enhances scalability and computational efficiency but, on the other hand, it brings major security and privacy challenges. The TLS 1.3 encryption used for data transmission is one method of protecting data, while secure storage protocols are another for data that is not in use. On-device anonymization guarantees data privacy by automatically blurring personally identifiable information like faces and license plates before it gets uploaded to the cloud. Model updates that are cryptographically signed are checked on the edge device before deployment to avoid any adversarial interference.

Real-time latency of under 100 ms is achieved to realize reliability via hardware optimization on both Jetson Nano and Raspberry Pi. Regular health checks of the sensors enable fault detection to happen fast, while if a problem occurs, the system will fall either in fail-safe or degraded mode. A hardware watchdog timer ensures recovery during software failure. In all, these measures make it possible to have secure, reliable, and real-time vehicle control in dynamic environments.

### Limitations

The proposed methodology, though highly effective in real-time object detection, has some drawbacks that might influence its practicality. To simulate the proposed method within the broader in the research landscape, it is important to note that while the CNN model indeed achieved high accuracy in tabular sensor fusion tasks, it is not directly comparable to compact vision-oriented models such as YOLOv8-n, MobileDet, or RT-DETR. Such state-of-the-art architectures are optimized for real-time image object detection, whereas the present CNN is designed for sequence-based trajectory prediction. The system relies on the processing power of edge devices such as Raspberry Pi and NVIDIA Jetson Nano, which may experience performance degradation when handling high resolution input. . Besides, the object detection performed by the cameras are very sensitive to bad weather conditions such as rain, fog, and poor lighting conditions that may lower object detection performance and impact the safety of vehicles. Ultrasonic sensors, while fine in measuring distance sensors are not good at sensing objects of irregular shape or non-reflective material, which can generate false obstacles. Cloud processing, although advantageous for model retraining and updates, suffers from latency issues due to network connectivity limitations, which can hinder timely decision-making in real-time systems..Model generalization also remains a challenge, as training on datasets such as COCO, KITTI, and other limited datasets may not fully represent real-world driving conditions. Variations in road curvature, environmental factors, and unforeseen objects can introduce inputs beyond the training distribution, leading to incorrect predictions. The high computational load of deep learning models like Faster R-CNN and YOLOv4 creates challenges in real-time computation on low-power edge devices, which limits scalability. Besides, it does not include complex traffic regulations, road signs, or adaptive driving patterns, making it less relevant to end-to-end autonomous vehicle solutions. In addition, the usage of multiple sensors, edge processing, and cloud computing has strong energy consumption, and this system is not suitable for low-power or cost-constrained applications. Furthermore, real-time decision-making with deep learning will require continuous model updating and calibration, which adds to maintenance work. Overcoming these limitations would require hardware selection optimization, improved sensor fusion techniques, improved algorithm efficiency, and higher adaptability to various real-world driving conditions.

## Ethics statements

None.

## CRediT authorship contribution statement

**Sumukh Chaurasia:** Methodology, Project administration. **Parambrata Sanyal:** Writing – original draft, Writing – review & editing. **Gagandeep Kaur:** Writing – review & editing, Supervision, Validation. **Satvik Barhanpure:** Methodology, Project administration. **Kshitij Bhele:** Writing – original draft, Validation. **Amol D. Wable:** Writing – review & editing. **Suhashini Awadhesh Chaurasia:** Supervision, Validation. **Rutuja Rajendra Patil:** Writing – review & editing. **Devika Verma:** Writing – review & editing.

## Declaration of competing interest

The authors declare that they have no known competing financial interests or personal relationships that could have appeared to influence the work reported in this paper.

## Data Availability

Data will be made available on request.
